# Diagnostic accuracy of metagenomic next-generation sequencing for cryptococcosis in immunocompetent and immunocompromised patients

**DOI:** 10.3389/fcimb.2022.997256

**Published:** 2022-10-20

**Authors:** Yi Su, Qing Miao, Na Li, Bi-jie Hu, Jue Pan

**Affiliations:** Zhongshan Hospital, Fudan University, Shanghai, China

**Keywords:** cryptococcosis, mNGS, immunocompetent, immunocompromised, diagnosis

## Abstract

**Objective:**

To compare the diagnostic accuracy of metagenomic next-generation sequencing (mNGS) for cryptococcosis in patients with different immune statuses with that of conventional detection.

**Methods:**

A total of 1442 specimens including 71 specimens from patients with cryptococcosis were analyzed in the study. The chi square test was used to screen the sensitivity and specificity of different detection methods for different specimen types. One-way ANOVA was used to compare the mNGS results with age, CD4, lymphocytes, IFN, IL-6, IL-2 and serum antigen assay.

**Results:**

The sensitivity of mNGS was 44.29% in *Cryptococcus* infection cases. The positive rate of mNGS results for bronchoalveolar lavage fluid (BALF, 87.50%) from immunocompromised patients was higher than that of BALF from immunocompetent patients (40.00%, p=0.04). The sensitivity of the serum *Cryptococcus* capsular antigen assay was 80.00% in immunocompetent patients and 96.42% in immunocompromised patients (p = 0.049). A positive rate of detection of *Cryptococcus* from mNGS was higher when cryptococcal antigen ≥1:160 (p=0.022) in immunocompromised patients. A positive rate of detection of *Cryptococcus* from mNGS was higher when lymphocyte counts were lower in both immunocompetent patients(p=0.017) and in immunocompromised patients(p=0.029).

**Conclusions:**

The sensitivity of mNGS is lower than that of serum cryptococcal antigen assay and histopathology in immunocompetent patients. However, BALF detection is recommend for immunocompromised patients compared with tissue and CSF. The positive mNGS result was correlated with lower lymphocyte counts, higher IL-2 and higher serum antigen assay in immunocompromised patients.

## Introduction

Cryptococcosis is an invasive fungal infection caused by *Cryptococcusneoformans* or *CryptococcusC. gattii* that has become increasingly prevalent in both immunocompetent and immunocompromised patients. Cryptococcosis is confirmed by positive culture, which can define the types of *Cryptococcus* and their antifungal susceptibility, but this method always takes 3-5 days. Positive results of cryptococcal antigen assays of serum, bronchoalveolar lavage fluid (BALF) or cerebrospinal fluid (CSF) and positive histopathologic results can help to quickly diagnose the infection but may be present false positive and false negative results.

Metagenomic next-generation sequencing (mNGS) is a technique that is increasingly used for the clinical diagnosis of infectious diseases, especially when the pathogen of the infectious disease cannot be cultured or detected normally (Han et al., 2019). Most studies have reported the use of mNGS in the diagnosis of bacterial and viral infections (Miao et al., 2018; Xie et al., 2019). In addition, many cases of rare pathogen infections detected by mNGS have been reported (Salzberg et al., 2016). However, few studies have analyzed the value of mNGS in the diagnosis of fungal infections, especially in patients with cryptococcosis. In our research, we summarized 1442 mNGS results and evaluate their diagnostic accuracy compared with that of conventional diagnosis methods.

Previous studies on cryptococcosis have mainly focused on cryptococcal infections in immunocompromised patients with central nervous system (CNS) infections. As a new detection method, for the first time, mNGS was analyzed and evaluated for its efficacy in both immunocompetent and immunocompromised patients, mainly for pulmonary cryptococcosis.

## Materials and methods

### Case series

Cases met the inclusion criteria if the patients were admitted to the Department of Infectious Disease from Zhongshan Hospital, Fudan University, from April 1, 2017, to January 31, 2021. This study is part of a research project that aims to detect pathogens from patients with clinically suspected infections using mNGS. The demographic, clinical feature, laboratory result, pathogenic finding, treatment and outcome data were extracted from the Hospital Information System of Zhongshan Hospital. The project was approved by the ethics committee of Zhongshan Hospital. All data were checked by two physicians (QM and YS), and a third researcher (JP) settled any difference in interpretation between the two primary reviewers.

### Case definition

Cases with an admitting diagnosis of suspected pulmonary infection or central nervous system infection were included in this retrospective analysis. Cryptococcus detection by mNGS (strict mapping read number (SMRN)≥1 or strict mapping read number genus (SMRNG)≥1) was defined as a positive result. Cryptococcosis includes confirmed cryptococcosis and clinical cryptococcosis.Confirmed cryptococcosis was defined as a positive *Cryptococcus* culture of a specimen from any site. Clinical cryptococcosis was defined byorpositive results on histopathologyor positive cryptococcal antigen assay, together with clinical or radiographic evidence of disease (Baddley et al., 2008). Immunocompromised hosts were considered to have at least one predisposing condition, including AIDS, liver cirrhosis, hematologic malignancy, malignant solid tumor, chronic steroid use and rheumatologic disease (rheumatoid arthritis, lupus, psoriatic arthritis, ankylosing spondylitis (AS), Sjogren’s syndrome, and inflammatory myopathy) (Brizendine et al., 2013; Lin et al., 2015).

### Laboratory diagnosis

#### Culture and identification

Cryptococcus was cultured from CSF, sputum, BALF, blood and tissue biopsy on Sabouraud Dextrose GC Agarplates(OXOID, Thermo Scientific, USA). Colonies are observed on solid agar plates after 48 to 72 hours incubation at 30°C to 35°C in aerobic conditions and will appear as opaque, white-to-cream colonies that may turn orange-tan or brown after prolonged incubation. Identifcation of fungal isolates was performed using the MALDI-Biotyper system.

##### Histopathology

Cryptococcus can be identified by histologic staining of tissues from the lung, skin, and other organs. The organism is observed as a yeast that reproduces by narrow-based budding. The yeast is identified by special stains that label the polysaccharide capsule including periodic acid-Schiff and Gomori methenamine silver. Biobsy was independently analysed by two trained examiners.

##### Serology

All blood, BALF and CSF samples were analysed using the Latex Cryptococcus Antigen Detection System (IMMY, Norman, USA). The test was performed according to the manufacturers’ instructions. Samples were independently analysed by two trained examiners, and no incongruences occurred. The results were read out in titre levels.

### mNGS procedure for specimens

A 0.5-3 mL sputum or BALF or CSFspecimen from the patients was collected according to standard procedures. Sputum was liquefied by addition of 0.1% DTT for 30 min at room temperature, and BALF or CSF was directly submitted to the next operation. Then, 1.5 mL microcentrifuge tubes with 0.5 mL of specimen and 1 g of 0.5 mm glass bead were attached to a horizontal platform on a vortex mixer and agitated vigorously at 2800-3200 RPM for 30 min. Next, 0.3 mL of specimen was separated into a new 1.5 mL microcentrifuge tube, and DNA was extracted using a TIANamp Micro DNA Kit (DP316, TIANGEN BIOTECH) according to the manufacturer’s recommendation. DNA could also be extracted directly from 300 μL CSF specimens and tissue specimens using the TIANamp Micro DNA Kit. The extracted DNA was sonicated to obtain 200-300 bp fragments (Bioruptor Pico protocols). Then, DNA libraries were constructed through end repair, adapter ligation and PCR amplification. We used an Agilent 2100 for quality control of the DNA libraries, and quality qualified libraries were sequenced by the BGISEQ-100 platform (Jeon et al., 2014). High-quality sequencing data were generated by removing low-quality and short (length<35 bp) reads, followed by computational subtraction of human host sequences mapped to the human reference genome (hg19) using Burrows–Wheeler alignment (Li and Durbin, 2009). The remaining data were classified by the removal of low-complexity reads and by simultaneous alignment to four microbial genome databases. The reference database for microbial classification was The Pathogens metagenomics Database (PMDB, v6.0), a commercial pathogen alignment database developed by PathoGenesis Pharmaceutical Technology (BGI-Shenzhen, Shenzhen), containing 4,945 whole genome sequence of viral taxa, 6,350 bacteral genomes or scaffolds, 1064 fungi related to human infection, and 234 parasites associated with human diseases. The PMDB contains genomes and genome assembles from NCBI, including Refseq and high quality genome assembles registered with NCBI.

### Statistical analysis of the data

Depending on the distribution of the data, categorical variables were described as percentages, and continuous variables were described as the mean and standard deviation. The chi square test was used to screen the sensitivity of mNGS from BALF, tissue and CSF, the specificity of mNGS from sputum, BALF, tissue, CSF and blood, the sensitivity of antigen assay from blood, BALF and CSF, the sensitivity of pathology from tracheoscopic, lung puncture and total and the sensitivity of culture from BALF, CSF and tissue. One-way ANOVA was used to compare the mNGS results withage ,CD4, lymphocytes, IFN, IL-6, IL-2,serum antigen assay. A probability (P) value <.05 implies a statistically significant difference. Statistical analyses were performed using SPSS software (version 23). The figures were constructed using GRAPHPAD PRISM 8.0.

## Results

### Specimens and patient characteristics

**Figure 1:**
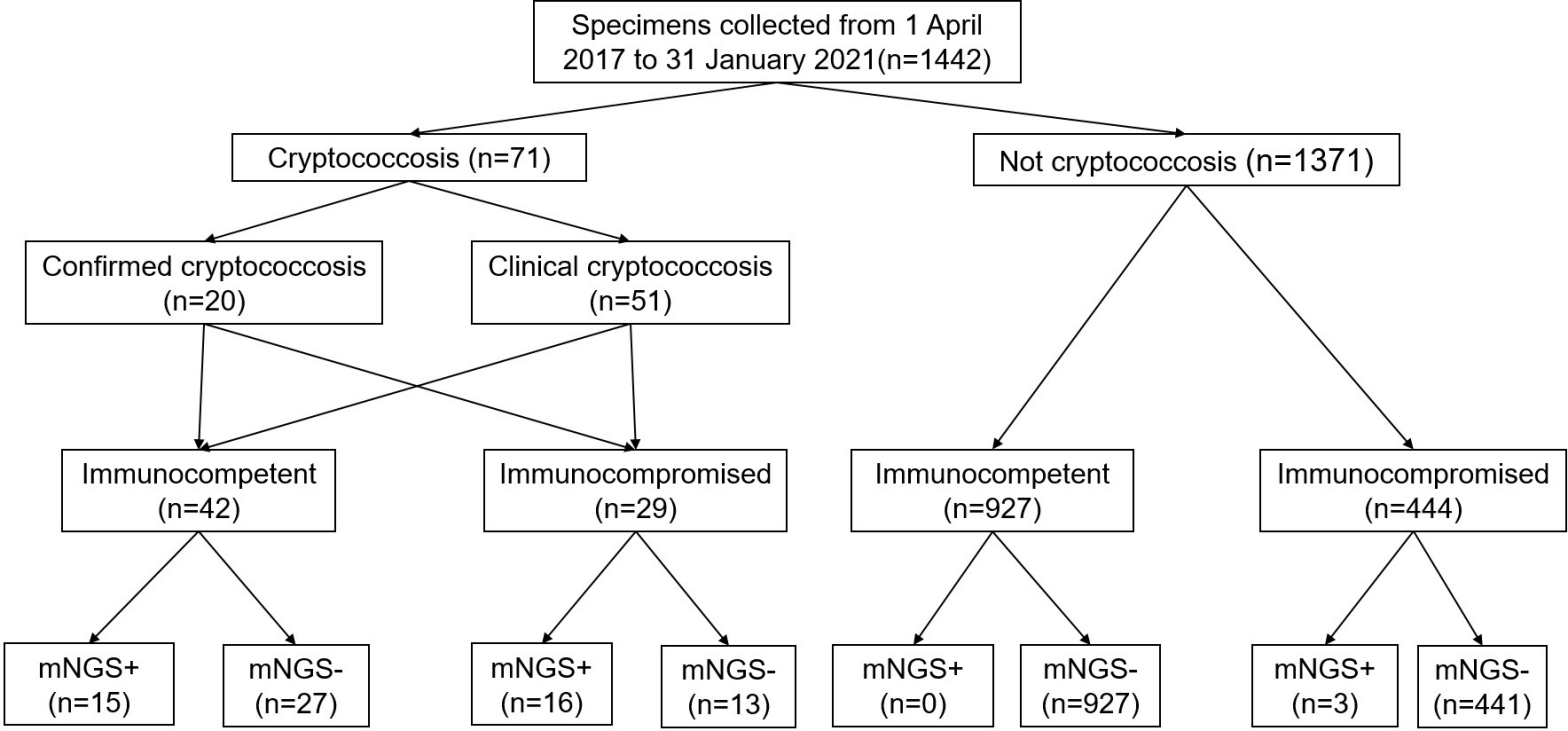
Flow chart of sample selection and classification. In total, 1442 specimens were divided into cryptococcosis and NOT cryptococcosis on the retrospective diagnosis of the corresponding patients, mNGS+ refers to strict mapping reads number(SMRN)2 for strict mapping reads number genus(SMRNG)21. mNGS, metagenomic next generation sequencing.

**Figure 2:**
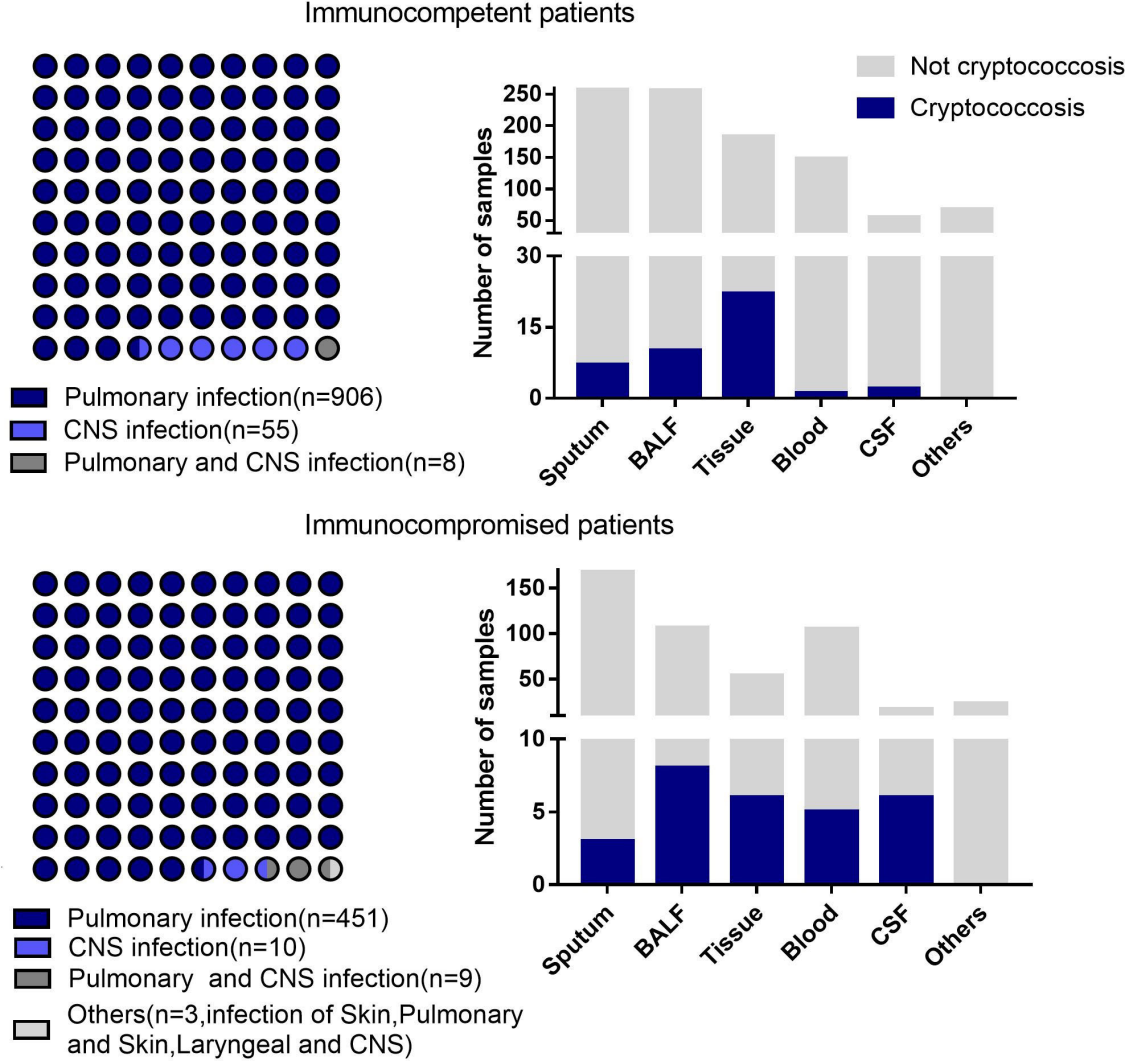
Disease distribution and different kinds of samples from the patients with different immune states. Others includes pleural effusion.

A total of 1442 specimens were included in the analysis, with 71 specimens from cryptococcosis cases and 1371 specimens from non-cryptococcosis cases (**Figure 1**). 61 (85.92%) specimens from 71 cryptococcosis cases were positive in antigen assay from blood, 20 (28.17%) were positive in culture, and 39 (54.93%) were positive in pathology. Of all 71 specimens from cryptococcosis cases before diagnosis, 19 specimens of cases did not use any antibacterial and antifungal drugs, 32 used antibacterial drugs, 8 used antifungal drugs, and 12 used both antibacterial and antifungal drugs. Of 20 specimens from confirmed *Cryptococcus* patients, 9 were from immunocompetent patients and 11 were from immunocompromised patients. Of 51 specimens from clinical *Cryptococcus* patients, 33 were from immunocompetent patients and 18 were from immunocompromised patients.Of all the specimens, 969 specimens were from immunocompetent patients, among which 530 (54.70%) specimens were from males with an average age of 53.79 ± 16.43 years and 439 (45.30%) specimens were from females with an average age of 54.05 ± 17.15 years. A total of 473 specimens were from immunocompromised patients, among which 251 (53.07%) specimens were from males with an average age of 55.90 ± 14.11 years and 222 (46.93%) specimens were from females with an average age of 59.36 ± 14.90 years. The distribution of different kinds of specimens from patients with different immune states is shown in **Figure 2**. SMRN of cryptococcosis cases ranged from 1 to 1333873 and the median was 40.5, SMRNG of cryptococcosis cases ranged from 1 to 1506102 and the median was 48.

### Sensitivity and specificity of mNGS from different specimens

**Figure 3:**
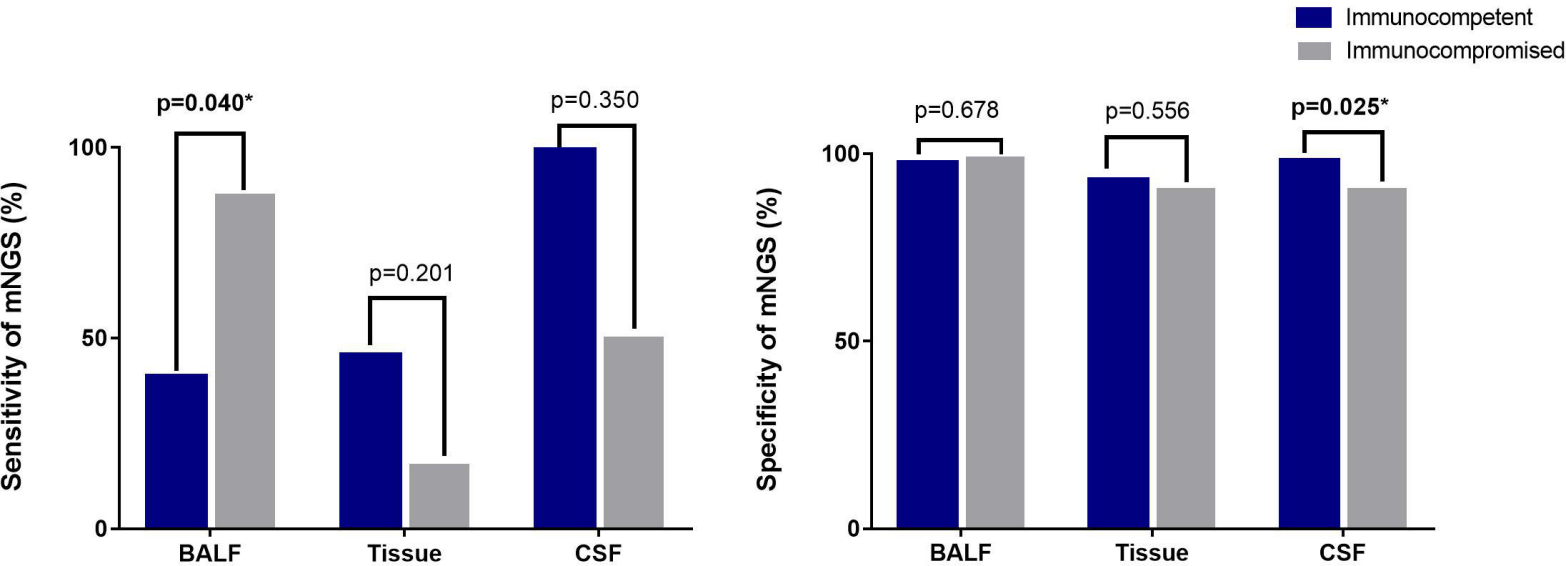
Sensitivity and specificity of mNGS from different samples by different immune statuses. The sensitivity of mNGS with sputum from 7 immunocompetent patients was 0%, and the positive rate of 3 sputum samples of immunocompromised patients was 33.33%. Sensitivity of mNGS from blood was not detected in immunocompetent patients. Two immunocompromised patients with disseminated cryptococcal infections were evaluated for mNGS of blood and the positive rate was 50%. The specificity of mNGS for sputum samples was 97.31% in immunocompetent patients and 98.79% in immunocompromised patients. The specificity of blood samples was 99,32% in immunocompetent patients and was 98.01% in immunocompromised patients. *means results has statistically significant.

The sensitivity of mNGS was 44.29% in Cryptococcus infection cases, and the specificity was 99.8%. The positive rate of mNGS of BALF from immunocompromised patients was higher than that of BALF from immunocompetent patients (p=0.04) (**Figure 3**).

### Sensitivity of different detection methods

**Figure 4:**
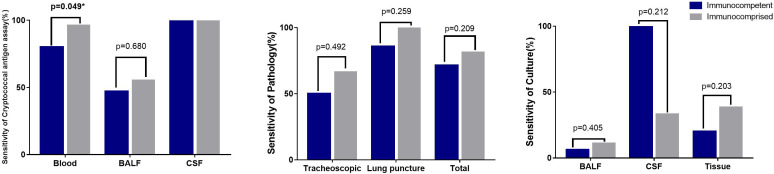
Sensitivity of different detection methods in different samples Sensitivity of 2 skin pathology from immunocomprised patients was 100%. The CSF culture in one immunocomptent patients of CNS cryptococcus infection was positive (100%), and 2 samples of CSF culture were positive from 6 samples of immunocomprised patients (33.3%).

The sensitivity of the serum cryptococcal antigen assay in immunocompetent patients was 80%, and that in immunocompromised patients was 96.42% (p = 0.049). Compared with those from immunocompromised patients, there were no significant sensitivity differences in the pathology, culture and tissue from immunocompetent patients (**Figure 4**).

### mNGS results with laboratory indexes

A positive rate of detection of *Cryptococcus* from mNGS was higher when cryptococcal antigen ≥1:160 (p=0.022) in immunocompromised patients. A positive rate of detection of *Cryptococcus* from mNGS was higher when lymphocyte counts were lower in both immunocompetent patients(p=0.017) and immunocompromised patients(p=0.029), as shown in **Table 1**.

**Table 1:** Relationship between positive mNGS results and laboratory indexes in patients with different immune statuses.

	Immunocompetent			Immunocompromised			
	mNGS+(n=15)	mNGS-(n=27)	p	mNGS+(n=16)	mNGS-(n=13)		p
Male/female	4/11	13/14	0.183	2/14		5/8	0.112
Age, years	46.67±14.11	44.52±14.49	0.645	56.06±11.59		54.69±15.95	0.791
CD4 (cells/µL)	518.35±255.66	621.19±282.26	0.265	291.07±257.18		505.85±369.51	0.083
Lymphocytes *10^^9^/L	1.31±0.81	1.81±0.46	0.017*	0.77±0.65		1.37±0.73	0.029*
IFN (8.1 pg/mL)	5.50±3.85	8.88±10.02	0.235	33.02±89.44		8.42±5.25	0.332
IL-6 (pg/mL)	4.36±6.21	7.69±25.51	0.635	4.64±4.34		7.38±5.74	0.162
IL-2>400 U/mL	327.42±286.53	382.92±268.67	0.185	437.28±405.83		648.15±432.12	0.006*
Serum antigen assay≥1: 160	184.13±355.61	128.15±279.32	0.293	716.00±894.91		253.23±689.05	0.022*

*means results has statistically significant. Bold values means results has statistically significant.

### Negative results from conventional detection methods and positive results from mNGS

From 3 patients diagnosed with community-acquired pneumonia, 2 sputum specimens and 1 BALF specimen for which *Cryptococcus* read number were detected were considered false positives. mNGS of 2 BALF specimens from immunocompetent cases detected the read number of *Cryptococcus*, but the serum antigen assay, pathology and culture were all negative. Combined with the patient’s clinical manifestations clinical diagnosis of *Cryptococcus* was considered (**Table 2**).

**Table 2:** List of patients with negative traditional detection method and positive mNGS results.

Sample No.	Age	Gender	Diagnosis	Immune status	Sample	SMRN	SMRNG	Treatment	Prognosis
***List of “false positive” samples in non-cryptococcus cases***									
S53	54	male	CAP	immunocompetent	sputum	0	1	levofloxacin	cure
S279	75	male	CAP	immunocompetent	sputum	9	8	levofloxacin	cure
S637	50	male	CAP		BALF	5	5	SMZ	cure
***List of “positive” samples in probable cryptococcus cases***									
S45	54	female	pulmonary infection	immunocompetent	BALF	8	8	fluconazole	cure
S372	51	male	pulmonary infection	immunocompetent	BALF	5	5	fluconazole	cure

## Discussion

Pulmonary fungal infections are one of the main problems in patients with immune disorders, and one of these infections is cryptococcosis, which must be differentiated from other fungal infections using precise laboratory methods (direct examination, culture, histopathology, antigens). As a new accurate diagnostic technique, the diagnostic value from mNGS in cryptococcosis patients with different immune states is worth exploring (Zarrinfar et al., 2012; Zanganeh et al., 2018; Kashefi et al., 2021). In our previous research, the values of mNGS outperformed those of culture, especially for Mycobacterium tuberculosis, viruses, anaerobes and fungi (Miao et al., 2018). This article confirms that the diagnostic value of mNGS in cryptococcosis is superior than that of culture.

The sensitivity of mNGS was only 44.29% in the diagnosis of Cryptococcus infections in our study, which was lower than that of cryptococcal antigen assay tests (>95% sensitivity) (Kabanda et al., 2014) and pathology (88% sensitivity in CT-guided percutaneous needle biopsy). The *Cryptococcus* cell wall is a two-layered structure composed of α-1,3-glucan, β-1,3 and β-1,6-glucan, chitin, chitosan, mannoproteins and other GPI-anchored proteins. The inner layer is mainly composed of β-glucans and chitin arranged as fibers parallel to the plasma membrane, and the outer layer contains α-glucan and β-glucan. The main reason for the low positive rate of *Cryptococcus* is that the nucleic acid cannot be released due to the thick cell wall and incomplete disruption of the *Cryptococcus* cell wall (Wang et al., 2018; Garcia-Rubio et al., 2020). Although mNGS has no advantage in sensitivity and cost, it is a fast method that takes less than 24 hours and reports microbial population identification. As cryptococcal antigen tests can be yield false negative results and biopsy could also fail, positive mNGS findings could remind clinicians of *Cryptococcus* infections and help to prove the diagnosis quickly (Wilson et al., 2011). Appropriate targeted antifungal drugs can be chosen immediately due to the speed of the test, and the microbial population can be identified.

In our research, three mNGS results were positive for *Cryptococcus* but were confirmed to be false positives. Since *Cryptococcus* is not a common contaminant or background fungus, a SMRN≥1 or a SMRNG≥1 should be reported in our rules. False positives may be caused by the operator’s misoperation, contamination by the same batch of specimens or other undetected reasons. On the other hand, the results of 2 cases were negative by traditional detection methods, and only mNGS results were positive. Since the clinical manifestation and imaging findings were consistent with cryptococcosis and pulmonary lesions were absorbed after anti-cryptococcal treatment, these positive results were considered true positives. Therefore, positive results in *Cryptococcus* detection from mNGS alone could not independently confirm cryptococcosis, and the clinical manifestations and results of conventional methods should be additionally considered for the diagnosis of cryptococcosis.

Positive mNGS results are quite different for different types of specimens. In our research, the positive rates of *Cryptococcus* detection in BALF and CSF specimens were higher than that in lung tissues, while the positive rate in sputum was the lowest. Since few blood specimens were analyzed, the corresponding rate could not be assessed in this research. In a previous study, 90 patients with focal pulmonary infections were analyzed, and the sensitivity of mNGS analysis in the pathological specimen group, transbronchial brushing group and bronchoalveolar lavage group was 90%, 66.7 and 50%, respectively (Chen et al., 2021). The positive rate of mNGS in tissues in radial probe endobronchial ultrasound guided transbronchial lung biopsy (TBLB) (78.7%) was significantly higher than that in the TBLB group (60.6%) (Li et al., 2020). Therefore, obtaining and analyzing appropriate specimens are recommended to improve the sensitivity of the mNGS detection.

Articles about the value of laboratory tests in patients with cryptococcosis with different immune states are relatively rare. The cryptococcal antigen assay is less sensitive in HIV-negative than in HIV-positive patients, and values of 56–83% have been reported in HIV-negative immunocompromised patients with *Cryptococcus* pneumonia (Singh et al., 2008). In our research, including the cryptococcal antigen assay, the sensitivity of the detection methods in different specimens from immunocompromised patients was higher than that from immunocompetent patients. Therefore, etiological detection should be employed more in immunocompromised patients since cryptococcal infections are preferentially observed immunocompromised patients.

Cytokines are key modulators of the immune response and play an essential role in the defense mechanism against fungal infections. Research on immune response patterns in septic shock patients indicated that increases in IFN-γ and IL-6 levels were significantly elevated in septic patients suffering from fungal infection, especially in the early course of the disease (O.Decker et al., 2017). A study of patients with cryptococcal meningitis reported that a CSF proinflammatory response consists of an interplay of Th1 (IFN-γ and IL-6), Th2 (IL-4 and IL-10) and Th17 cytokines (IL-17A) and has been shown to be highly predictive of increased macrophage activation, rapid infection clearance and consequently improved survival (Jarvis et al., 2015). Another study also reported that IFN-γ could improve host immune responses against cryptococcal infection in HIV-infected patients (Jarvis et al., 2012). In our study, specimens with increasing cytokine levels (IL-2>400) yielded more positive mNGS results. Therefore, mNGS-based *Cryptococcus* findings correspond with plasma cytokine levels, which means that in patients with increasing cytokine levels, the mNGS-based approach revealed signs of a *Cryptococcus* infection prior to the culture-based finding, providing the opportunity to initiate targeted antifungal therapy in infected patients.

## Conclusion

The sensitivity of mNGS is lower than that of serum cryptococcal antigen assay and histopathology in immunocompetent patients.BALF detection is recommend for immunocompromised patients compared with tissue and CSF. The positive mNGS result was correlated with lower lymphocyte counts, higher IL-2 and higher serum antigen assay in immunocompromised patients

## Data availability statement

The datasets presented in this study can be found in online repositories. The names of the repository/repositories and accession number(s) can be found below: EMBL-EBI, PRJEB55094.

## Ethics statement

The studies involving human participants were reviewed and approved by Ethics Committee of Zhongshan Hospital Fudan University. The patients/participants provided their written informed consent to participate in this study. Written informed consent was obtained from the individual(s) for the publication of any potentially identifiable images or data included in this article.

## Author contributions

YS conceived and designed the work that led to the submission, QM played an important role in interpreting the results, NL acquired data, B-JH revised the manuscript, and JP revised the manuscript. All authors contributed to the article and approved the submitted version.

## Funding

This work was supported by grants from the National Key Research and Development Program of China(2021YFC 2300400),the Clinical Research Plan of SHDC (SHDC2020CR2031B) and the Fund of Zhongshan Hospital (2021ZSFZ15).

## Conflict of interest

The authors declare that the research was conducted in the absence of any commercial or financial relationships that could be construed as a potential conflict of interest.

## Publisher’s note

All claims expressed in this article are solely those of the authors and do not necessarily represent those of their affiliated organizations, or those of the publisher, the editors and the reviewers. Any product that may be evaluated in this article, or claim that may be made by its manufacturer, is not guaranteed or endorsed by the publisher.
